# IPEX as a Result of Mutations in FOXP3

**DOI:** 10.1155/2007/89017

**Published:** 2007-11-04

**Authors:** Hans J. J. van der Vliet, Edward E. Nieuwenhuis

**Affiliations:** ^1^Department of Medical Oncology, Vrije Universiteit Medical Center, De Boelelaan 1117, 1081 HV Amsterdam, The Netherlands; ^2^Department of Pediatric Gastroenterology, Erasmus Medical Center, Sophia Childrens' Hospital, 3000 GE Rotterdam, The Netherlands

## Abstract

Immunodysregulation, polyendocrinopathy, enteropathy, X-linked (IPEX) syndrome is a rare disorder caused by mutations in the FOXP3 gene that result in the defective development of $\text{CD}4^{+}\text{CD}25^{+}$ regulatory T cells which constitute an important T cell subset involved in immune homeostasis and protection against autoimmunity. Their deficiency is the hallmark of IPEX and leads to severe autoimmune phenomena including autoimmune enteropathy, dermatitis, thyroiditis, and type 1 diabetes, frequently resulting in death within the first 2 years of life. Apart from its clinical implications, IPEX illustrates the importance of immunoregulatory cells such as $\text{CD}4^{+}\text{CD}25^{+}$ regulatory T cells.

Immunodysregulation, polyendocrinopathy, enteropathy, X-linked (IPEX) syndrome is a rare disorder that was first described in 1982 [1]. It usually presents in the first few months of life with dermatitis, mostly manifested by eczema, although psoriatiform lesions and nodular pemphigoid have also been described. Other symptoms include cachexia and growth retardation as a result of the combination of autoimmune enteropathy, usually manifested by severe watery diarrhea, type 1 diabetes mellitus (DM), and chronic inflammation with excessive cytokine production [2]. In essence, IPEX is the result of a severe dysregulation of the immune system that can also lead to thyroiditis, hypothyroidism, autoimmune hemolytic anemia, recurrent infections, and membranous nephropathy [2, 3]. Without treatment, children usually die in the first 2 years of life due to sepsis or failure to thrive.

**FOXP3**

IPEX is an X-linked recessive disorder that is caused by mutations in the *FOXP3* gene that is located on the X-chromosome (Xq11.23-Xq13.3) and consists of 11 coding exons [4, 5]. After the initial discovery of this gene defect in *scurfy* mice, which develop a disease highly homologous to that of patients with IPEX, mutations were demonstrated in the orthologous human gene [6]. IPEX might however be genetically more heterogeneous than initially presumed as a few patients with IPEX were found not to carry mutations in *FOXP3*. Furthermore, in 1 family of patients that included an affected female, IPEX was linked to an autosomal locus [3, 7]. In these cases, IPEX could be the result of regulatory or conditional mutations outside *FOXP3* coding regions. Indeed, Bennett et al. identified a mutation in a region following the final coding exon, resulting in a reduction in FOXP3 mRNA expression [8].

To date, 20 mutations in *FOXP3 * have been identified in patients with IPEX—most of which result in a change in the amino acid sequence in the DNA-binding domain of the FOXP3 protein [9–12]. Figure 1 shows a schematic view of FOXP3 with locations of missense mutations indicated by arrows. Mutations in the winged-helix/forkhead (FKH) domain interfere with nuclear import and DNA binding, critical to FOXP3 repressor activity, while mutations in the leucine zipper impair FOXP3 dimerization, and hence DNA binding [12, 13]. Functional domains within the N-terminal half of FOXP3 have been identified and shown to be involved in general transcriptional repression and repression of NFAT-mediated transcription [12, 13]. It has also been suggested that some mutations can lead to an increase in the length of the carboxy terminus of FOXP3 resulting in a change in the three-dimensional structure and the position of the winged helix, or lead to a decrease in the stability of FOXP3 mRNA [5, 8, 11].

The most important characteristic of FOX proteins is the “forkhead box,” which consists of a sequence of 80–100 amino acids that together form a DNA-binding motif. The capacity to bind to DNA allows FOX proteins to regulate the expression of genes involved in cell growth, proliferation, differentiation, and lifespan. *FOXP3* codes a 48-kD protein (431 amino acids) from the forkhead/winged-helix transcription factor family, and it is highly expressed in CD4^+^CD25^+^ regulatory T cells [14, 15]. This T cell subset is involved in limiting the immune response of other cells, for example, conventional T cells. As in conventional T cells, activation of CD4^+^CD25^+^ regulatory T cells leads to the induction of the transcription factor NFAT (nuclear factor of activated T cells). In case of conventional T cells, T cell receptor signaling in combination with costimulation will result in binding of NFAT to AP-1 (activator protein 1), thereby inducing transcription of genes involved in T cell activation. In CD4^+^CD25^+^ regulatory T cells, however, NFAT preferentially binds to FOXP3 and results in the transcription of a whole different set of genes, resulting in immune suppression (Figure 2) [16].

**CD4^+^CD25^+^ regulatory T cells**

Immunosuppressive CD4^+^CD25^+^ regulatory T cells constitute a small subset (5–10%) of CD4^+^ T (helper) cells that develop in the thymus. They are characterized by the expression of the interleukin (IL) 2 receptor *α*-chain (CD25), cytotoxic T lymphocyte-associated antigen 4 (CTLA-4), and glucocorticoid-induced TNF receptor (GITR). The most specific molecular marker of CD4^+^CD25^+^ regulatory T cells, which is functionally involved in immune suppression, is the FOXP3 protein. In the absence of FOXP3, CD4^+^CD25^+^ regulatory T cells do not develop. The mechanisms mediating the immunosuppressive effects in vivo have not been fully elucidated. Several studies suggest immunosuppression to be cell contact-dependent, while other studies demonstrate that suppression can also be cell contact-independent, for example, via the production of the immunosuppressive cytokines IL-10 and TGF-β, preferential IL-2 consumption by CD4^+^CD25^+^ regulatory T cells, or direct lysis of T cells via perforin and granzymes (Figure 3) [10, 11, 14]. Noteworthily, the initiation of immunosuppression by CD4^+^CD25^+^ regulatory T cells requires antigen exposure, but suppressive effects can extend through bystander immunosuppression. CD4^+^CD25^+^ regulatory T cells have been demonstrated to suppress various types of immune responses, including autoimmune, antimicrobial, and antitumor immune responses (Figure 3) [14, 17].

**IPEX**

The incidence of IPEX is not known, but it is likely to be rare although unfamiliarity with the disease might be a contributing factor [3]. Although the inheritance pattern of IPEX is X-linked recessive in case of mutations in *FOXP3* (with no reported female patients, and asymptomatic female carriers of the gene mutation), the disease might be genetically more heterogeneous than initially presumed as a few patients with IPEX were found not to carry mutations in *FOXP3* . Furthermore, in 1 family of patients that included an affected female, IPEX was suggested to be linked to an autosomal locus [3, 7]. Symptoms usually start in the perinatal period or in early childhood, but they can also first arise in adulthood. First symptoms usually include type 1 DM and secretory diarrhea or ileus. Additional symptoms include eczema, thrombocytopenia, (Coombs-positive) anemia, lymphadenopathy, and thyroiditis/hypothyroidism. Renal insufficiency, caused by glomerulopathy and interstitial nephritis, arthritis, and ulcerative colitis have also been described in several cases. Patients with IPEX can be immunocompromised as a result of the defect in immunoregulation, autoimmune neutropenia, and immunosuppressive therapy. This can result in severe infectious complications and sepsis (especially catheter related) [1–3, 9].

**Diagnosis**

An important prerequisite for diagnosing IPEX is clinical suspicion. In this regard, the family history can be of value, though one has to realize that symptoms of the disease can vary within one family, for example, due to differences in *FOXP3* regulating elements or differences in modifying genes, environment, or therapy. Laboratory evaluation shows, depending on the exact presentation of disease, signs of type 1 DM, enteropathy, hypothyroidism, and/or cytopenias. No consistent changes in the function or number of “conventional” T cells, granulocytes, complement system, or immunoglobulin concentrations have been described. Histopathology shows the absence of normal mucosa of the small bowel and sometimes of the colon with diffuse infiltration of inflammatory cells in the lamina propria and submucosa. Inflammatory infiltrates can also occur in other organs including pancreas, skin, and kidney [3]. In the blood, autoantibodies can be detected against erythrocytes, thyroid, and pancreas [18]. Definitive diagnosis is based on DNA analysis showing mutations in the *FOXP3 * gene, though immunocytochemical analysis of FOXP3-expressing cells in bowel biopsies might provide a more rapid screening tool [19]. As mentioned, to date, 20 mutations in *FOXP3* have been identified [9, 10]. In patients with a mild presentation of IPEX, a mutation in *FOXP3 * was recently identified that did not interfere with protein expression but that did result in a partial defect in CD4^+^CD25^+^ regulatory T cell immunosuppression [10]. One of these patients presented at the age of 4 months with dehydration as a result of severe enteritis, hemolytic anemia, and eczema. Remarkably, symptoms disappeared without immunosuppressive therapy and the patient, now 2 years old, only incidentally complains of mild eczema. This patients' brother carries the same mutation but he has not shown any symptom of the disease (until at least 3.5 years of age).

**Therapy**

Apart from supportive care (including parenteral nutrition, blood transfusions, and treatment of diabetes), immunosuppressive therapy and bone marrow transplantation have shown efficacy. As a result of the rarity of the syndrome, therapies have not been studied in large-scale clinical trials, but in either small series or case reports. Various immunosuppressive medications have been administered successfully in IPEX including high-dose steroids, cyclosporin A, tacrolimus, methotrexate, infliximab, and rituximab [1], reviewed in [3, 20–24]. Unfortunately, these immunosuppressants are usually only partially effective and the dose is limited in large part by infectious complications and toxicity [3, 20]. Sirolimus was suggested to be more effective as it resulted in a sustained (followup up to 5 years) suppression of systemic inflammatory, gastrointestinal, and dermatologic symptoms of IPEX in 3 patients. However, these patients received sirolimus in combination with azathioprine or methotrexate and received prior treatment with steroids and tacrolimus [20]. A promising form of therapy seems bone marrow transplantation, although the transplantation protocol probably needs further optimization. In several patients, allogenous transplantation resulted in a rapid and sustained reduction in symptoms of enteropathy, eczema, and diabetes which was accompanied by a reduction in the concentration of autoantibodies directed against pancreas [18, 25]. In several of these patients, a donor/acceptor chimerism was found, suggesting that a limited number of healthy donor leukocytes are sufficient to suppress the auto-immune process [18, 25]. Noteworthily , transplanted patients do not always have an uncomplicated followup as one patient died after a disease-free interval of 29 months as a result of a lymphoproliferative hemophagocytic syndrome, while two other patients died from infectious complications, probably due to earlier long-term immunosuppressive treatment, after an initial improvement in their clinical condition [3, 18]. Importantly, recently beneficial results have been described in 4 patients who were treated with bone marrow transplantation after a “reduced-intensity” conditioning regimen using alemtuzumab, fludarabine, and melphalan [26]. In all 4 patients, colitis and allergies subsided after transplantation, and transplantation-related complications were mainly infectious in the first phase, though in 1 patient, acute and subsequent chronic graft-versus-host disease developed, which necessitated immunosuppressive therapy with tacrolimus [19]. It seems reasonable to consider transplantation in an early phase of the disease as this might reduce the risk of infectious complications of chronic immunosuppressive therapy, and curtail the autoimmune damage to endocrine organs.

## CONCLUDING REMARKS

IPEX illustrates the central regulatory role of regulatory T cells in the immune system. Importantly, apart from the severe defects in CD4^+^CD25^+^ regulatory T cells that result in IPEX, other, perhaps more subtle, changes in the CD4^+^CD25^+^ regulatory T cell population can contribute to the pathogenesis of disease. In various autoimmune syndromes, for example, quantitative or qualitative defects in CD4^+^CD25^+^ regulatory T cells have been reported [2]. In contrast, in various forms of cancer, an increase in the number of CD4^+^CD25^+^ regulatory T cells was noted and related to downregulation of antitumor immune responses and poor prognosis [27]. In this situation, a reduction in the number of CD4^+^CD25^+^ regulatory T cells could be beneficial, as demonstrated by a study in which CD4^+^CD25^+^ regulatory T cell depletion enhanced the antitumor immune response in renal cell cancer [28]. Clearly, a further increase in knowledge on the biology of regulatory T cells will likely result in new therapeutic strategies for a specific set of immune-mediated disorders.

## Figures and Tables

**Figure 1:**
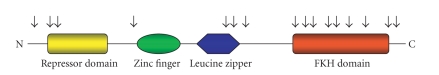
Schematic representation of human FOXP3. Arrows indicate the location of identified missense point mutations in patients with IPEX.

**Figure 2:**
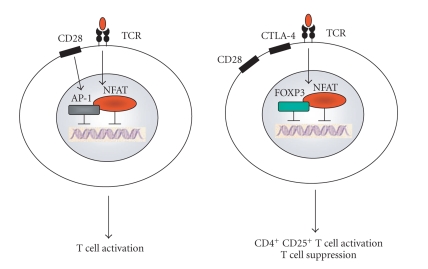
FOXP3 regulates NFAT-mediated gene transcription in CD4^+^CD25^+^ regulatory T cells. In both conventional T cells (left) and CD4^+^CD25^+^ regulatory T cells (right), T cell receptor-(TCR-) mediated activation upregulates the expression of the transcription factor NFAT. Upon TCR stimulation and CD28-mediated costimulation, NFAT binds to AP-1 in conventional T cells but to FOXP3 in CD4^+^CD25^+^ regulatory T cells. As a consequence, activation results in the transcription of a different set of genes in both cell types.

**Figure 3:**
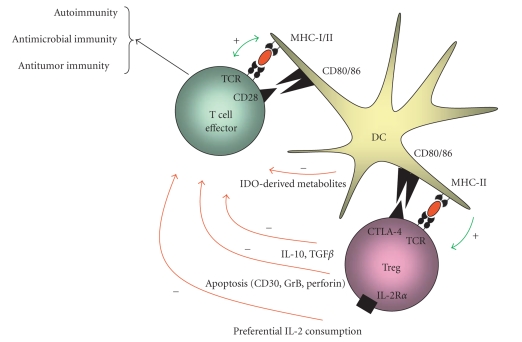
Schematic representation of the regulation of the function of effector T cells by CD4^+^CD25^+^ regulatory T cells. Upon antigen-specific activation by dendritic cells (DCs), CD4^+^CD25^+^ regulatory T (Treg) cells can suppress immune responses through (a) preferential consumption of IL-2 by CD4^+^CD25^+^ regulatory T cells instead of effector T cells, (b) induction of effector T cell apoptosis via CD30/CD30L interactions or perforin/granzyme B (GrB), (c) production of immunosuppressive cytokines IL-10 and TGF-β by CD4^+^CD25^+^ regulatory T cells, and (d) production of immunosuppressive tryptophane metabolites as a result of upregulation of indoleamine-2,3-dioxygenase (IDO) in DC.
